# Mindful Jazz and Preferred Music Interventions Reduce Pain Among Patients With Chronic Pain and Anxiety: A Pilot Randomized Controlled Trial

**DOI:** 10.7759/cureus.80485

**Published:** 2025-03-12

**Authors:** Sean D Young, Josh Kim, Adam Hanley

**Affiliations:** 1 Emergency Medicine, University of California Irvine, Irvine, USA; 2 School of Information and Computer Sciences, University of California Irvine, Irvine, USA; 3 Informatics, University of California Irvine, Irvine, USA; 4 Psychology, Florida State University, Tallahasee, USA

**Keywords:** : anxiety, chronic pain management, listening to music, mindfulness-based interventions, music intervention

## Abstract

Background: A mindfulness-based intervention (MBI) focused on listening to music might reduce chronic pain and provide a new approach to overcoming challenges from traditional MBIs (e.g., breathing). Due to the potential unpredictability and unfamiliarity of jazz, an MBI focused on listening to improvisational jazz music might be a particularly efficacious pain reduction intervention. This pilot study explores whether mindfully listening to music, including jazz, can reduce pain-related outcomes.

Methods: Chronic musculoskeletal pain (CMP) participants (n=30 per group, N = 120 total) were enrolled online between 12/7/2023 and 2/8/2024. Participants were randomly assigned to one of four groups in a 2 (Mindful Music Listening/Intervention vs. Music Education/Control) X 2 (Preferred Music (choose their own music genre) vs Jazz (assigned to listen to improvisational jazz)) experiment, for a total of four groups (Mindful Jazz, Mindful Music, Jazz Education, and Music Education). Patients in each group were provided with training in either mindful listening to music (Intervention groups) or music education (Control groups) and given four sets of weekly recordings related to their group for daily listening/practice. Patients completed online surveys on pain-related outcomes (e.g., pain catastrophizing, pain intensity, and anxiety) pre- and post-training (immediate outcomes), and throughout a four-week period (longer-term outcomes). The main outcomes analyses compared the intervention and control groups, with secondary sub-analyses among participants who listened to at least 2/3 of their recordings (10 minutes), and among those who experienced a clinically meaningful (20%) reduction in pain.

Results: Mindful Jazz and Mindful Music (Intervention) participants reported significantly less pain intensity (p < 0.001) and pain unpleasantness (p < 0.001) immediately after the training relative to the Jazz Education and Music Education (Control) participants. Mindful Jazz participants also reported a significant reduction in anxiety compared to the Jazz and Music Education groups (p < 0.05). Throughout the four-week period, Mindful Jazz participants reported less pain intensity relative to both control groups (Jazz and Music Education); Mindful Music participants reported significantly less pain intensity relative to only the Jazz Education participants. Mindful Jazz participants reported a >20% decrease in pain intensity more frequently than Jazz Education (Χ^2^=48.71, p<0.001), Music Education (Χ^2^=65.13, p<0.001), and Mindful Music (Χ^2^=8.74, p=0.003) participants. Similarly, among the instances when a participant listened to at least 10 minutes of their audio recording, the proportion who achieved a >20% decrease in pain intensity differed significantly ((Χ^2^=84.03, p<0.001): Jazz Education, 29%; Music Education, 26%, Mindful Jazz, 50%; Mindful Music 41%).

Conclusion: Mindfully listening to music can help to reduce pain-related outcomes. Both music education (i.e., music listening without mindfulness training) and mindfully listening to music (i.e., listening with mindfulness training) helped to decrease pain and anxiety from baseline to follow-up. However, mindful listening reduced pain to a greater amount compared to music education, suggesting that mindfully listening to music is a more impactful pain reduction intervention compared to listening without mindfulness training. Future research is warranted with a larger sample.

## Introduction

Chronic musculoskeletal pain (CMP), including spinal pain and osteoarthritis, is the leading cause of years lived with disability worldwide and the most expensive health condition in the United States [1-3]. Compounding these concerns, opioid analgesics remain the primary pharmacological treatment for CMP [4,5]. Troublingly, it is estimated that almost 30% of chronic pain patients receiving long-term opioid therapy develop opioid misuse [6], which can further lead to addiction and overdose [7-9]. CMP patients therefore need better, non-addictive treatment options.

Music listening interventions, and mindful breathing interventions, have both been found, independently, to reduce pain and related symptomology. For example, meta-analytic results suggest that listening to music can reduce acute and chronic pain [10,11], pain-related emotional distress, as well as use of pain medication [10]. Music interventions have demonstrated these positive effects on pain across settings, including surgical, in-patient, and community settings [10-12]. Importantly, due to the ubiquity of music-- especially freely available online music--music interventions are easily accessible and highly scalable, making it important to further study their potential impact on health.

Similarly, many studies, including multiple meta-analyses, have found that mindfulness-based interventions (MBI) reduce chronic pain and pain-related symptomology [13,14]. For example, in a randomized controlled trial on 250 chronic pain patients, an MBI significantly reduced chronic pain-related functional interference (p<0.001, d=.58) and pain intensity (p=0.003, d=0.36) through the nine-month follow-up, with 50% of patients reporting clinically significant pain reductions [15]. Numerous studies have been conducted and found consistent results with MBI’s demonstrating similar positive results on pain reduction.

However, despite the success of these traditional MBIs, many of them are inaccessible for CMP patients due to both formatting and content barriers. Greater adherence to MBIs is associated with better clinical outcomes, but traditional mindfulness practices (e.g., teaching patients to mindfully attend to their breathing) can be difficult, boring, or even uncomfortable for some CMP patients [16-23]. For example, some MBI studies of broader patient populations have had dropout rates as high as 47% [20]. One study found that more than 25% of participants said their MBI experience was unpleasant and/or boring [21]. Mindfulness apps have attempted to address this problem by making mindful breathing practices more accessible to users, but these apps still struggle to retain users in the practice. For example, despite mindfulness apps being the most commonly downloaded wellness apps [24], user retention is only 50% two days after installation and approximately 5% thirty days after installation [25]. Developing MBI formats that teach CMP patients to practice mindfulness in ways that are more familiar, gentle, and consistent for their lifestyles (e.g., mindfully listening to music) may increase mindfulness practice engagement and allow more CMP patients to benefit from MBIs.

Combining these two interventions, music listening and mindfulness-based interventions, by teaching people how to mindfully listen to music, may help to leverage the successes of both interventions while also addressing the limitations of MBIs by allowing patients to mindfully attend to something easier and more comfortable than their breathing (i.e., music). 

Although most music listening studies have allowed participants to choose their own preferred styles of music, certain styles, or components (e.g., rhythms, chord progressions) of music, such as improvisational jazz, might be particularly effective when incorporated into a mindful listening intervention to reduce pain. For example, unfamiliar music may be particularly well-suited for mindful music listening by calling the listener’s attention to the present moment, curiosity, and openness. Jazz is one of the least familiar genres of music to most Americans [26-27], and therefore a reasonable genre to include when testing this theory on the impact of mindfully listening to unfamiliar music. A specific style of jazz, improvisational jazz, may be especially relevant for testing this hypothesis in a study as the often rapid and unpredictable musical changes invite the listener to stay in the present moment, much as the musicians must be when improvising. For example, although there are jazz “standards” that have the same familiar beginning melody (e.g., “My Favorite Things”), musicians play these improvisational jazz songs completely different each time depending on their immediate emotions. Improvisational jazz is, therefore, a truly present-minded experience for the musician (and possibly for the listener too, if they can be taught to attentively follow the music’s improvisation). Taken together, mindfully listening to jazz might be especially helpful in reducing patients’ pain and anxiety, and increasing their present-mindedness. However, little to no research has explored this topic.

This pilot study seeks to assess whether being trained to mindfully listen to music, particularly jazz, compared to not receiving mindful listening training, leads to reduced pain and anxiety among patients with chronic pain. Because most music listening studies have allowed participants to choose their own style of music, we have created two music conditions, participant’s choice/preferred music and improvisational jazz. This study was also designed to test the broader impact of “mindful music listening” compared to traditional music listening (with added music education, as a control for the amount of time spent in training) on pain-related outcomes.

## Materials and methods

Methods

A total of 120 participants with chronic pain were recruited using paid online advertisements (e.g., Facebook, and Instagram). Those who clicked on the ad were routed to an online screening website to screen for eligibility. Inclusion criteria were: self-reported 18 years of age or older, little to no experience listening to jazz, lives in the United States, English-proficient adult with a chronic pain diagnosis, has a pain rating of five or greater in average pain on a 0-10 numeric rating scale in the preceding week, has had pain for at least three months and for at least 15 days in the preceding 30 days. Exclusion criteria were: currently using or having used prescription opioids for more than three months (to reduce interactions with other forms of medical treatment), current cancer diagnosis, or experience with mindfulness. Those found preliminarily eligible received an online written information sheet (informed consent) and completed an online baseline survey on demographics and chronic pain-related outcomes (e.g., pain catastrophizing, pain self-efficacy, anxiety).

Participants who completed the baseline survey were then contacted by a research associate to further confirm eligibility by making sure they were real people and not a bot or an invalid responder [28]. They were then randomly assigned and called to schedule training. Participants were randomly assigned to one of four groups in a 2 × 2 design: (1) Mindful Music (Intervention) vs. Music Education (Control), and (2) Preferred Music (participants chose their own music genre) vs. Jazz (participants were assigned to listen to improvisational jazz), for a total of four groups (Mindful Jazz, Mindful Music, Jazz Education, and Music Education). This 2 x 2 design allowed us to compare mindfully listening to jazz to the other groups but also allowed us to combine mindfully listening to jazz with mindfully listening to one’s preferred musical genre and test whether mindfully listening to any type of music might reduce pain compared to listening to it without training in mindfulness. The training sessions for each group were 20 minutes in length, conducted in an individual format, and were led by a trained research associate via a secure video-conferencing platform.

The music education groups were designed to be similar to traditional research on music and health, by allowing participants to listen to music. Because the intervention groups taught participants how to mindfully listen to music, we also included a time-matched "teaching" portion for the music education groups where the research associate read a script to teach participants about music history and education. Participants then watched an educational video about music (Music Education Group, https://www.science.edu/acellus/wp-content/uploads/2023/07/Music-Appreciation-Kindergarten-Eileen-1.mp4?_=1) or specific to improvisational jazz (Jazz Education Group, https://youtu.be/BMgKXbtQwoo) that was designed to inspire them to want to listen to music.

The Mindful Jazz and Mindful Music groups were similar to the Jazz and Music Education groups but included instructions in mindfulness training instead of music education. Those in the Mindful Jazz and Mindful Music groups were read a time-matched script that was designed to teach them about mindfulness and how to mindfully listen to music. Participants in the mindfulness groups saw a video created by a study team member that provided participants with audio-guided instruction for how to mindfully listen to a song (“Thrushes in Parc Eric Satie” by David Patterson for the preferred music groups), (“Have You Met Ms. Jones” by the Oscar Peterson Trio for the jazz groups). 

Participants in all four groups were asked to listen to at least 10 minutes of music each day for the first four weeks after finishing their training. No upper limit to the amount of daily music listening was set. To encourage standardized listening experiences, participants in the groups listening to their preferred style of music were provided with four playlists (one for each follow-up week) of songs from their preferred genre (e.g., rock, hip hop, classical). Similarly, participants in the jazz groups were provided with four playlists (one for each follow-up week) of jazz songs. Jazz playlists included instrumental versions of songs such as “Take the A Train,” “St. Thomas,” and “Kind of Blue.” All playlists were approximately 15 minutes in length. Each day, before and after listening to their playlist, participants were asked to complete single-item measures of pain intensity, pain unpleasantness, and anxiety, scored on an 11-point numeric rating scale. After four weeks, all participants were invited to complete a final survey. Participants were paid in Amazon gift cards for completing the baseline ($20) and final ($25) survey. This study was deemed self-exempt by the University of California, Irvine Institutional Review Board (IRB). This study was registered on clinicaltrials.gov (NCT05979012) prior to enrollment of the first participant (first submitted July 28, 2023).

Analyses were thematically grouped to examine the primary variables of interest at two different time scales: (1) from immediately before to immediately after intervention exposure (i.e., testing for immediate outcomes occurring the same day as they received their training), and (2) during the four-week follow-up period (i.e., follow-up outcomes). First, generalized linear mixed modeling (GLMM) was used to analyze the immediate clinical outcomes: pain intensity, pain unpleasantness, and anxiety. In each of these mixed models, outcome variables were regressed (Jazz Education vs. Music Education vs. Mindful Jazz vs. Mindful Music) and the respective baseline values in accordance with the classical analysis of the covariance approach for analyzing clinical trial outcomes [29]. Next, t-tests were used to examine within-group changes in the immediate outcome variables. A responder analysis was also performed by calculating the proportion of participants with a ≥20% (i.e., minimally clinically important, based on prior research) improvement in pain intensity from baseline [30]. A chi-square test was used to determine whether the number of responders differed by group. Finally, bivariate correlation analysis was used to examine relations among residualized change scores created for each clinical and mechanistic outcome.

Second, GLMM was used to analyze the four-week follow-up outcomes: daily pain intensity, pain unpleasantness, and anxiety during independent, “at home” listening. In each of these mixed models, the outcome variable (e.g., pain intensity after listening to the provided music playlist) was regressed (Jazz Education vs. Music Education vs. Mindful Jazz vs. Mindful Music), time (Day 0 through Day 28), a condition X time interaction term, the respective baseline value (e.g., pain intensity before listening to the provided music playlist), and time spent listening to the playlist to account for “dosage”. Additionally, t-tests were used to examine within-group changes in the follow-up outcome variables for instances when at least 10 minutes of the provided playlist (i.e., 2/3 of the recording) was listened to by a participant. A responder analysis was conducted using the same criteria as above (i.e., ≥20% decrease in pain intensity). 

All tests and confidence intervals were two-sided and statistical significance was defined as a p-value less than 0.05. The path model was conducted in AMOS version 29 (IBM Corp., Armonk, NY) all other statistical analyses were conducted in SPSS version 29 (IBM Corp., Armonk, NY).

## Results

All study participants (n=120) were enrolled between 12/7/2023 and 2/8/2024. Participants’ demographic and clinical characteristics did not differ significantly by group (See Table 1). The majority of participants were White (74%) and female (80%). Moderate pain levels (mean =7.22, SD=1.21) were reported in the week prior to intervention exposure and 84% of participants reported experiencing chronic pain for more than a year. Regardless of group, all participants completed their entire intervention.

**Table 1:** Demographic Differences by Group. Statistical tests used: one-way ANOVA, chi-squared test. Average pain was measured by a 0 to 10 numeric rating scale item adapted from the Brief Pain Inventory.

Variables	Jazz Education	Music Education	Mindful Jazz	Mindful Music	Test Statistic	p
Primary Pain Condition					Χ^2^=18.23	0.44
Back	12 (40%)	11 (37%)	11 (37%)	11 (37%)		
Fibromyalgia	7 (23%)	2 (7%)	4 (13%)	2 (7%)		
Head	-	3 (10%)	4 (13%)	5 (17%)		
Joint	8 (27%)	8 (27%)	4 (13%)	6 (20%)		
Neck	-	2 (7%)	3 (10%)	1 (3%)		
Nerve	2 (7%)	2 (7%)	-	2 (7%)		
Other	1 (3%)	2 (7%)	4 (13%)	3 (10%)		
Average Pain, mean (SD)	7.23 ± 1.31	7.07 ± 1.23	7.33 ± 1.12	7.23 ± 1.22	F=0.25	0.86
Pain Duration > 1 year, n (%)	27 (90%)	23 (77%)	26 (87%)	25 (83%)	Χ^2^=4.87	0.56
Age	46.47 (11.83)	40.20 (12.05)	46.70 (14.17)	45.83 (14.78)	F=1.63	0.19
Sex					Χ^2^=4.08	0.67
Female	25 (83%)	24 (80%)	26 (87%)	25 (83%)		
Male	5 (17%)	6 (20%)	3 (10%)	5 (17%)		
Intersex	-	-	1 (3%)	-		
Gender					Χ^2^=3.00	0.81
Female	24 (80%)	22 (73%)	26 (87%)	24 (80%)		
Male	5 (17%)	5 (17%)	3 (10%)	5 (17%)		
Other	1 (3%)	3 (10%)	1 (3%)	1 (3%)		
Sexual Orientation					Χ^2^=12.53	0.40
Bisexual	2 (7%)	5 (17%)	6 (20%)	-		
Gay	2 (7%)	1 (3%)	1 (3%)	1 (3%)		
Heterosexual	23 (77%)	20 (67%)	21 (70%)	27 (90%)		
Questioning	1 (3%)	2 (7%)	-	-		
Other	2 (7%)	2 (7%)	2 (7%)	2 (7%)		
Hispanic or Latino	3 (10%)	5 (17%)	5 (17%)	4 (13%)	Χ^2^=3.84	0.70
Race					Χ^2^=11.39	0.72
American Indian or Alaska Native	-	1 (3%)	-	-		
Asian	1 (3%)	1 (3%)	1 (3%)	2 (7%)		
Black or African American	3 (10%)	6 (20%)	3 (10%)	6 (20%)		
White	23 (79%)	21 (70%)	25 (83%)	20 (67%)		
Unknown	-	1 (3%)	-	-		
Two or more	2 (7%)	-	1 (3%)	2 (7%)		
Education					Χ^2^=14.82	0.67
Upper secondary	-	-	-	1 (3%)		
Diploma or equivalent (GED)	5 (17%)	2 (7%)	3 (10%)	2 (7%)		
Some college/certificate	8 (27%)	10 (33%)	12 (40%)	7 (23%)		
Vocational/trade school	2 (7%)	2 (7%)	2 (7%)	1 (3%)		
Bachelor’s degree	5 (7%)	8 (27%)	5 (17%)	11 (37%)		
Some graduate or professional school	2 (7%)	1 (3%)	4 (13%)	1 (3%)		
Completed graduate or professional School	8 (27%)	7 (23%)	4 (13%)	7 (23%)		
Employment					Χ^2^=8.91	0.71
Full-time employment	14 (47%)	13 (43%)	8 (28%)	14 (47%)		
Not employed	11 (37%)	9 (30%)	15 (52%)	8 (27%)		
Part-time employment	4 (13%)	5 (17%)	4 (13%)	7 (23%)		
Contractor/consultant	1 (3%)	2 (7%)	1 (3%)	-		
Student	-	1 (3%)	1 (3%)	1 (3%)		
Relationship Status					Χ^2^=13.50	0.33
Divorced	6 (20%)	2 (7%)	9 (30%)	8 (27%)		
Married	10 (33%)	12 (40%)	7 (23%)	11 (37%)		
Never married	11 (37%)	16 (53%)	11 (37%)	10 (33%)		
Separated	2 (7%)	-	1 (3%)	-		
Widowed	1 (3%)	-	2 (7%)	1 (3%)		
Household Size	2.33 ± 1.32	2.83 ± 1.64	2.57 ± 1.61	2.60 ± 1.40	F=0.56	0.64
Household income					Χ^2^=14.00	0.30
< $24,999	10 (33%)	6 (20%)	6 (20%)	2 (7%)		
$25,000 to $49,999	5 (17%)	5 (17%)	8 (27%)	6 (20%)		
$50,000 to $99,999	10 (33%)	13 (43%)	7 (23%)	12 (40%)		
> $100,000	5 (17%)	4 (13%)	6 (20%)	9 (30%)		
Prefer not to answer	-	2 (7%)	3 (10%)	6 (3%)		

Immediate clinical outcomes

Significant between-group effects on pain intensity and pain unpleasantness were observed. Mindful Jazz and Mindful Music participants reported significantly less pain intensity and pain unpleasantness immediately after the intervention relative to the Jazz Education and Music Education participants. Mindful Jazz participants also reported a significant reduction in anxiety compared to the Jazz and Music Education Groups in pairwise between-group comparisons (Tables 2-3). The remaining contrasts were not significant. No effect of group was observed for anxiety.

**Table 2:** Estimated marginal means by treatment group for immediate outcomes. Statistical test used: generalized linear mixed modeling

Variables	Jazz Education	Music Education	Mindful Jazz	Mindful Music	F	p
Pain Intensity	5.50 (5.05 to 5.94)	5.46 (5.01 to 5.90)	4.51 (4.06 to 4.95)	4.54 (4.10 to 4.99)	6.03	< 0.001
Pain Unpleasantness	5.09 (4.55 to 5.64)	4.87 (4.32 to 5.42)	3.72 (3.18 to 4.27)	3.71 (3.16 to 4.26)	7.04	< 0.001
Anxiety	3.34 (2.79 to 3.90)	3.25 (2.69 to 3.80)	2.41 (1.85 to 2.96)	2.94 (2.38 to 3.50)	2.26	0.09

**Table 3:** Table 3. Pairwise between group comparisons for immediate outcomes. Variables are presented as pairwise between group comparisons Statistical test used: t-test * p<0.05 **<0.01 ***p< 0.001

Variables	Condition	Music Education	Mindful Jazz	Mindful Music
Pain Intensity	Jazz Education	0.04 (-0.59 to 0.67)	0.99** (0.36 to 1.61)	0.95** (0.33 to 1.59)
	Music Education	-	0.95** (0.32 to 1.58)	0.91** (0.29 to 1.54)
	Mindful Jazz	-	-	-0.03 (-0.66 to 0.60)
Pain Unpleasantness	Jazz Education	0.22 (-0.46 to 1.00)	1.37*** (0.60 to 2.15)	1.38*** (0.61 to 2.16)
	Music Education	-	1.15** (0.37 to 1.93)	1.16** (0.38 to 1.94)
	Mindful Jazz	-	-	0.01 (-0.76 to 0.79)
Anxiety	Jazz Education	0.09 (-0.69 to 0.88)	0.93* (0.15 to 1.72)	0.40 (-0.39 to 1.19)
	Music Education	-	0.84* (0.05 to 1.62)	0.31 (-0.48 to 1.09)
	Mindful Jazz	-	-	-0.53 (-1.32 to 0.26)

Significant within-group effects were observed for all variables of interest. Effect size estimates were generally in the large range for the Mindful Jazz and Mindful Music groups; Effect size estimates were generally in the medium-to-large range for the Jazz Education and Music Education groups (Table 4).

**Table 4:** Within-group changes in the primary variables of interest for immediate outcomes. Statistical test used: t-test Δ = change in value from pre- to post-intervention, d= standardized effect size

Variable	Condition	Pre-intervention	Post-intervention	Δ	t	p	d
Pain Intensity	Jazz Education	6.10 ± 1.47	5.53 ± 1.72	-0.57	3.80	< 0.001	0.69
	Music Education	6.03 ± 1.40	5.43 ± 1.72	-0.60	2.98	0.006	0.55
	Mindful Jazz	5.93 ± 1.89	4.40 ± 1.99	-1.53	5.59	< 0.001	1.02
	Mindful Music	6.17 ± 1.49)	4.63 ± 1.90	-1.53	5.87	< 0.001	1.07
Pain Unpleasantness	Jazz Education	5.83 ± 1.64	5.10 ± 1.85	-0.73	3.43	0.002	0.63
	Music Education	5.40 ± 1.96	4.57 ± 2.08	-0.83	2.93	0.006	0.54
	Mindful Jazz	5.93 ± 2.23	3.80 ± 2.07	-2.13	6.25	< 0.001	1.14
	Mindful Music	6.13 ± 1.70	3.93 ± 2.12	-2.20	7.05	< 0.001	1.29
Anxiety	Jazz Education	5.00 ± 2.77	3.50 ± 2.52	-1.50	3.86	< 0.001	0.70
	Music Education	4.60 ± 2.71	3.17 ± 2.52	-1.43	4.87	< 0.001	0.89
	Mindful Jazz	5.17 ± 2.72	2.67 ± 1.90	-2.50	6.98	< 0.001	1.28
	Mindful Music	4.17 ± 2.49	2.60 ± 1.79	-1.57	5.00	< 0.001	0.91

The number of participants that achieved a ≥20% decrease in pain intensity differed significantly by group (Χ^2^=13.35, p=0.004): Jazz Education, n=9 (30%); Music Education, n=8 (27%), Mindful Jazz, n=20 (67%); Mindful Music, n=16 (53%). There was a significantly higher percentage of Mindful Jazz participants that reported a ≥20% decrease in pain intensity relative to Jazz Education (Χ^2^=8.08, p=0.004) and Music Education (Χ^2^=9.64, p=0.002) participants. Additionally, significantly more Mindful Music participants reported a ≥20% decrease in pain intensity than Music Education participants (Χ^2^=8.08, p=0.004). The remaining contrasts were not significant.

Follow-up clinical outcomes

Significant between-group effects on daily pain intensity and pain unpleasantness during the one-month follow-up period were observed. Mindful Jazz and Mindful Music participants reported significantly less pain intensity and pain unpleasantness relative to the Jazz Education participants. Additionally, the Mindful Jazz participants reported significantly less pain intensity relative to both control groups (Jazz Education and Music Education participants, Tables 5-6). Although not significant, Mindful Jazz participants reported less pain intensity and anxiety compared to Mindful Music participants. The remaining contrasts were not significant. No effect of the group was observed for anxiety.

**Table 5:** Estimated marginal means by treatment group for follow-up outcomes. Statistical test used: generalized linear mixed modeling

Variable	Jazz Education	Music Education	Mindful Jazz	Mindful Music	F	p
Pain intensity	4.72 (4.37 to 5.07)	4.61 (4.26 to 4.96)	4.05 (3.68 to 4.42)	4.16 (3.80 to 4.53)	3.63	0.013
Pain unpleasantness	4.41 (4.01 to 4.80)	4.00 (3.61 to 4.40)	3.62 (3.20 to 4.05)	3.60 (3.19 to 4.01)	3.61	0.013
Anxiety	3.51 (3.12 to 3.89)	3.35 (2.97 to 3.73)	2.98 (2.57 to 3.39)	3.02 (2.62 to 3.42)	1.01	0.39

**Table 6:** Pairwise between group comparisons for follow-up outcomes. Variables are presented as pairwise between group comparisons Statistical test used: t-test * p<0.05; ** p<0.01

Variable	Conditions	Music Education	Mindful Jazz	Mindful Music
Pain Intensity	Jazz Education	0.11 (-0.38 to 0.60)	0.67* (0.16 to 1.18)	0.55* (0.05 to 1.06)
	Music Education	-	0.56* (0.05 to 1.07)	0.45 (-0.06 to 0.95)
	Mindful Jazz	-	-	-0.11 (-0.63 to 0.41)
Pain Unpleasantness	Jazz Education	0.40 (-0.16 to 0.96)	0.78** (0.21 to 1.36)	0.81** (0.24 to 1.38)
	Music Education	-	0.38 (-0.20 to 0.96)	0.41 (-0.17 to 0.98)
	Mindful Jazz	-	-	0.03 (-0.57 to 0.62)
Anxiety	Jazz Education	0.15 (-0.39 to 0.70)	0.53 (-0.03 to 1.09)	0.49 (-0.07 to 1.04)
	Music Education	-	0.37 (-0.19 to 0.93)	0.33 (-0.22 to 0.89)
	Mindful Jazz	-	-	-0.04 (-0.61 to 0.53)

During the follow-up period, there was no difference in the number of times a participant listened to >10 minutes of their audio recording by group (Χ^2^=2.14, p=0.55): Jazz Education, k=456 (70%); Music Education, k=439 (72%), Mindful Jazz, k=350 (69%); Mindful Music, k=409 (70%).

Among the instances when a participant listened to 10 minutes of their audio recording (Table 7), the proportion that achieved a ≥20% decrease in pain intensity differed significantly by group (Χ^2^=84.03, p<0.001): Jazz Education, 182 listens out of a total of 456 listens (i.e., 29%); Music Education, 154 listens out of a total of 439 listens (26%), Mindful Jazz, 234 listens out of a total of 350 listens (50%); Mindful Music, 233 listens out of a total of 409 listens (41%). Mindful Jazz participants reported a ≥20% decrease in pain intensity more frequently than Jazz Education (Χ^2^=48.71, p<0.001), Music Education (Χ^2^=65.13, p<0.001), and Mindful Music (Χ^2^=8.74, p=0.003) participants. Mindful Music participants also reported a ≥20% decrease in pain intensity more frequently than Jazz Education (Χ^2^=17.59, p<0.001) and Music Education (Χ^2^=28.95, p<0.001) participants. The remaining contrasts were not significant.

**Table 7:** Within group changes in the primary variables of interest for follow-up outcomes. Statistical test used: t-test Δ = change in value from pre- to post-intervention; d= standardized effect size

Variable	Condition	Pre-intervention	Post-intervention	Δ	T	p	d
Pain Intensity	Jazz Education	5.27 ± 1.69	4.56 ± 1.82	-0.72	14.25	< 0.001	0.67
	Music Education	4.95 ± 2.37	4.31 ± 2.30	-0.64	11.26	< 0.001	0.54
	Mindful Jazz	5.82 ± 2.29	4.28 ± 2.27	-1.54	17.37	< 0.001	0.93
	Mindful Music	5.18 ± 2.23	4.04 ± 2.36	-1.14	16.17	< 0.001	0.80
Pain Unpleasantness	Jazz Education	4.99 ± 2.08	4.18 ± 2.05	-0.81	13.40	< 0.001	0.63
	Music Education	4.51 ± 2.45	3.67 ± 2.28	-0.85	11.88	< 0.001	0.57
	Mindful Jazz	5.34 ± 2.72	3.65 ± 2.36	-1.69	16.64	< 0.001	0.89
	Mindful Music	4.94 ± 2.42	3.48 ± 2.25	-1.45	17.98	< 0.001	0.90
Anxiety	Jazz Education	4.33 ± 2.44	3.40 ± 2.34	-0.93	16.38	< 0.001	0.77
	Music Education	4.11 ± 2.69	3.25 ± 2.47	-0.87	11.88	< 0.001	0.57
	Mindful Jazz	4.54 ± 2.88	2.78 ± 2.20	-1.75	15.89	< 0.001	0.85
	Mindful Music	3.77 ± 2.71	2.51 ± 2.20	-1.25	17.38	< 0.001	0.86

## Discussion

Results suggest that mindfully listening to music can help to reduce pain, and anxiety, and increase measures of mindfulness among patients with chronic pain. As expected, based on prior research, listening to music after music education (i.e., a music listening intervention) and mindfully listening to music (i.e., a mindful listening intervention) both helped to decrease patients’ pain and anxiety from baseline to follow-up. However, both groups of mindful listening (Mindful Music and Mindful Jazz) reduced pain to a greater amount compared to music education, suggesting that mindfully listening to music is a more impactful pain reduction intervention compared to just listening to music without training in how to mindfully attend to it.

This study builds on prior research on music and health [12] as well as mindfulness and health, in a number of ways. First, most randomized controlled pain trials involving music used music in the intervention group, but did not have music involved in a control group. This design can study whether (or not) music impacted pain, but is limited in not understanding what about the music intervention condition led to the change. Was the change caused by the music itself, the way users interacted with the music, their preference for the music, or something else? In this study, all four groups included music, which allowed us to conclude that listening to music after learning how to mindfully listen to music, in particular, jazz music, helps to reduce pain more than listening to music without learning how to listen mindfully. The present design also builds upon research on the barriers to practicing mindfulness by creating a new (potentially more accessible and engaging) method for practicing mindfulness by mindfully listening to music. Finally, the present study builds on other types of non-pharmacological interventions, such as online behavior change community interventions, that have been used to help patients suffering from chronic pain [31-33] and mental health issues [34], by offering a new type of intervention integrating mindfulness and music. 

Importantly, mindful listening to music may help to increase engagement for patients who are less willing or able to participate in other types of pain-reduction interventions, including traditional MBIs [16-23]. The entertaining nature of music, along with its ubiquity (i.e., music heard throughout the day, in cars/buses/trains, elevators, on the television, phone, etc) make it so that people can easily sustain a mindful listening practice throughout different parts of the day, every day, leading to potentially high engagement and retention in mindful listening practice. We expect that not all participants in the Mindful Jazz and Mindful Music groups practiced daily mindfulness when listening to the music. There may be multiple reasons why people were still not engaged in mindful listening, but the high engagement rates found and the relationship between engagement and pain intensity shed light on the need for future research to study how mindful listening may reduce pain. 

Interestingly, and providing support for the argument for unfamiliar music, or at least improvisational jazz, Mindful Jazz participants who had listened to at least 2/3 of their audio recording were significantly more likely to have a 20% reduction in pain intensity compared to all other groups, including Mindful Music participants. Additionally, although not significant perhaps due to the small sample in this pilot, we found a trend suggesting that mindfully listening to jazz reduces anxiety and pain intensity compared to mindfully listening to one’s preferred choice in music. As improvisational jazz musicians are “living in the moment” by spontaneously composing their music, learning how to listen to these notes might help listeners to similarly live in the moment and reduce anxiety. However, due to the small sample size from this pilot study, as well as potential confounders in combining multiple types of improvisational jazz songs and components of those songs together, future research is needed to further explore this hypothesis. Specifically, it is unclear whether and how certain types of improvisational jazz songs or components within the songs might be more effective than preferred or other styles of music for inducing this present-minded listening and reducing anxiety.

This study was limited by a small sample size (pilot study), lack of prior research on mindful listening interventions, jazz, and health, and having recruited a convenience sample of online participants. Given the high prevalence of internet/social media use in the United States, and the benefits of being able to easily and rapidly scale and implement studies using online samples and online interventions, we do not believe the online sample is a major limitation. Future research with a larger sample will help to better highlight and confirm the findings from this pilot study. Additional research is needed on mindful listening interventions, as well as the potential benefits of listening to improvisational music (including identifying the specific components/theoretical framework for this pathway). Future research can also include patients on opioid therapy to better understand how the intervention might affect their therapy progress and needs. 

## Conclusions

Mindfully listening to music may help to reduce pain, decrease anxiety, and increase mindfulness among patients with chronic pain. Due to the lack of effective non-pharmacological treatments for chronic pain, identifying alternative treatments for pain reduction are highly needed, especially those that are highly accessible, scalable, and have the potential for high engagement, such as mindful listening. However, prior to implementation, future research is needed in a larger sample to more definitively assess the impact of mindful listening on pain. Additional research is needed to better understand the mechanisms behind how different components of music (e.g., components of improvisational jazz) might strengthen a mindful listening intervention. 
